# Supplementary material for: Integrated transcriptomic and neuroimaging brain model decodes biological mechanisms in aging and Alzheimer’s disease
Source: eLife. 2021 May 18;10:e62589. doi: 10.7554/eLife.62589 (PMC8131100; doi:10.7554/eLife.62589)
Supplement: Supplementary file 1. [file elife-62589-supp1.docx]

**Supplementary File 1**: Main demographic characteristics of the included ADNI subjects.

| **Variable** | **HC**  **(N=151)** | **EMCI (N=161)** | **LMCI (N=113)** | **AD (N=35)** | **Stable HC (N=113)** | **AD + converters (N=129)** |
| --- | --- | --- | --- | --- | --- | --- |
| Female | 76(50.3%) | 68(42.2%) | 51(45.1%) | 16(45.7%) | 59(52.2%) | 58(45%) |
| Mean age (years) | 74(5.5) | 70.1(6.8) | 71.7(7.1) | 74.7(8.1) | 73.7(5.6) | 73.2(7.1) |
| Mean education (years) | 16.5(2.7) | 16.3(2.7) | 16.2(2.9) | 15.2(2.6) | 16.8(2.5) | 15.8(2.7) |

Data are number (%) or mean (std).
