# Supplementary material for: Integrated transcriptomic and neuroimaging brain model decodes biological mechanisms in aging and Alzheimer’s disease
Source: eLife. 2021 May 18;10:e62589. doi: 10.7554/eLife.62589 (PMC8131100; doi:10.7554/eLife.62589)
Supplement: Supplementary file 3. [file elife-62589-supp3.docx]

**Supplementary File 3:** Brain regions used in this study. A total of 144 regions (72 regions each in both hemispheres) were derived from Julich and Brodmann’s atlases.

| **Number** | **Julich Atlas** | **Number** | **Brodmann's Atlas** |
| --- | --- | --- | --- |
| 1 | hOc1 | 44 | Brodmann's area 1 |
| 2 | hOc2 | 45 | Brodmann's area 2 |
| 3 | hOc4d | 46 | Brodmann's area 3 |
| 4 | hOc3d | 47 | Brodmann's area 4 |
| 5 | hOc3v | 48 | Brodmann's area 5 |
| 6 | hOc4v | 49 | Brodmann's area 6 |
| 7 | 1 | 50 | Brodmann's area 7 |
| 8 | 2 | 51 | Brodmann's area 10 |
| 9 | †3a | 52 | Brodmann's area 11 |
| 10 | 3b | 53 | Brodmann's area 17 |
| 11 | FG1 | 54 | †*Brodmann's area 18 |
| 12 | FG2 | 55 | Brodmann's area 19 |
| 13 | Brodmann's area 37 | 56 | Brodmann's area 24 |
| 14 | Te1 | 57 | Brodmann's area 25 |
| 15 | Te2 | 58 | Brodmann's area 26 |
| 16 | Brodmann's area 20 | 59 | Brodmann's area 27 |
| 17 | Brodmann's area 21 | 60 | Brodmann's area 29 |
| 18 | Brodmann's area 22 | 61 | †*Brodmann's area 30 |
| 19 | Brodmann's area 36 | 62 | Brodmann's area 32 |
| 20 | Brodmann's area 38 | 63 | Brodmann's area 34 |
| 21 | 5L | 64 | Brodmann's area 35 |
| 22 | 5M | 65 | Brodmann's area 39 |
| 23 | PGa | 66 | Brodmann's area 40 |
| 24 | PGp | 67 | †Brodmann's area 41 |
| 25 | PFt | 68 | Brodmann's area 42 |
| 26 | PFm | 69 | Brodmann's area 43 |
| 27 | p24ab | 70 | Brodmann's area 44 |
| 28 | p32 | 71 | Brodmann's area 45 |
| 29 | Brodmann's area 23 | 72 | Brodmann's area 48 |
| 30 | 6 |  |  |
| 31 | 4p |  |  |
| 32 | Brodmann's area 8 |  |  |
| 33 | Brodmann's area 9 |  |  |
| 34 | Fp1 |  |  |
| 35 | Fp2 |  |  |
| 36 | Fo1 |  |  |
| 37 | 44 |  |  |
| 38 | 45 |  |  |
| 39 | Brodmann's area 46 |  |  |
| 40 | Brodmann's area 47 |  |  |
| 41 | 7A |  |  |
| 42 | CA+dentate |  |  |
| 43 | Brodmann's area 28 |  |  |

* Region excluded from the left hemisphere

† Region excluded from the right hemisphere
