# Supplementary material for: Integrated transcriptomic and neuroimaging brain model decodes biological mechanisms in aging and Alzheimer’s disease
Source: eLife. 2021 May 18;10:e62589. doi: 10.7554/eLife.62589 (PMC8131100; doi:10.7554/eLife.62589)
Supplement: Supplementary file 5. [file elife-62589-supp5.docx]

**Supplementary File 5**: Identified molecular pathways underlying AD progression.

| **Pathway** | **No of genes** |
| --- | --- |
| CCKR signaling map | 8 |
| Inflammation mediated by chemokine and cytokine signaling pathway | 6 |
| Apoptosis signaling pathway | 5 |
| Gonadotropin-releasing hormone receptor pathway | 5 |
| Heterotrimeric G-protein signaling pathway-Gi alpha and Gs alpha mediated pathway | 3 |
| FAS signaling pathway | 3 |
| p38 MAPK pathway | 3 |
| Enkephalin release | 3 |
| Beta3 adrenergic receptor signaling pathway | 2 |
| Beta2 adrenergic receptor signaling pathway | 2 |
| Beta1 adrenergic receptor signaling pathway | 2 |
| 5HT4 type receptor mediated signaling pathway | 2 |
| Angiogenesis | 2 |
| Alzheimer disease-presenilin pathway | 2 |
| Ubiquitin proteasome pathway | 2 |
| Wnt signaling pathway | 2 |
| N-acetylglucosamine metabolism | 2 |
| Cytoskeletal regulation by Rho GTPase | 2 |
| Histamine H2 receptor mediated signaling pathway | 2 |
| Cell cycle | 2 |
| B cell activation | 2 |
| Cortocotropin releasing factor receptor signaling pathway | 2 |
| Axon guidance mediated by netrin | 1 |
| Axon guidance mediated by Slit/Robo | 1 |
| Metabotropic glutamate receptor group III pathway | 1 |
| JAK/STAT signaling pathway | 1 |
| Interleukin signaling pathway | 1 |
| Interferon-gamma signaling pathway | 1 |
| 5HT2 type receptor mediated signaling pathway | 1 |
| Coenzyme A biosynthesis | 1 |
| 5HT1 type receptor mediated signaling pathway | 1 |
| Insulin/IGF pathway-protein kinase B signaling cascade | 1 |
| Insulin/IGF pathway-mitogen activated protein kinase kinase/MAP kinase cascade | 1 |
| Huntington disease | 1 |
| Heterotrimeric G-protein signaling pathway-rod outer segment phototransduction | 1 |
| p53 pathway | 1 |
| p53 pathway feedback loops 2 | 1 |
| Heterotrimeric G-protein signaling pathway-Gq alpha and Go alpha mediated pathway | 1 |
| p53 pathway by glucose deprivation | 1 |
| O-antigen biosynthesis | 1 |
| Xanthine and guanine salvage pathway | 1 |
| Transcription regulation by bZIP transcription factor | 1 |
| Thyrotropin-releasing hormone receptor signaling pathway | 1 |
| Toll receptor signaling pathway | 1 |
| Ras Pathway | 1 |
| Adenine and hypoxanthine salvage pathway | 1 |
| T cell activation | 1 |
| Oxytocin receptor mediated signaling pathway | 1 |
| Endothelin signaling pathway | 1 |
| EGF receptor signaling pathway | 1 |
| Parkinson disease | 1 |
| DNA replication | 1 |
| PI3 kinase pathway | 1 |
| Opioid proopiomelanocortin pathway | 1 |
| PDGF signaling pathway | 1 |
| Opioid prodynorphin pathway | 1 |
| Oxidative stress response | 1 |
| Opioid proenkephalin pathway | 1 |
| Cholesterol biosynthesis | 1 |
